# The cannabinoid receptor-1 gene interacts with stressful life events to increase the risk for problematic alcohol use

**DOI:** 10.1038/s41598-022-08980-w

**Published:** 2022-03-23

**Authors:** Lisa Bornscheuer, Andreas Lundin, Yvonne Forsell, Catharina Lavebratt, Philippe A. Melas

**Affiliations:** 1 Department of Public Health Sciences, Stockholm University, 10691 Stockholm, Sweden; 2 Center for Molecular Medicine, L8:00, Karolinska University Hospital, 17176 Stockholm, Sweden; 3 Department of Global Public Health, Karolinska Institutet, 17177 Stockholm, Sweden; 4 Department of Molecular Medicine and Surgery, Karolinska Institutet, 17176 Stockholm, Sweden; 5 Center for Psychiatry Research, Department of Clinical Neuroscience, Karolinska Institutet and Stockholm Health Care Services, 11364 Stockholm, Sweden

**Keywords:** Addiction, Genetic association study

## Abstract

Problematic alcohol use is a major contributor to the global burden of death and disabilities, and it represents a public health concern that has grown substantially following the COVID-19 pandemic. The available treatment options remain limited and to develop better pharmacotherapies for alcohol misuse we need to identify suitable biological targets. Previous research has implicated the brain’s endocannabinoid system (ECS) in psychiatric and stress-related outcomes, including substance use and habituation to repeated stress. Moreover, genetic variants in the cannabinoid-1 receptor gene (*CNR1*; *CB1R*) have been associated with personality traits, which are in turn predictors of substance use disorders. To date, however, no human genome-wide association study has provided evidence for an involvement of the ECS in substance use outcomes. One reason for this ECS-related “missing heritability” may be unexamined gene-environment interactions. To explore this possibility, we conducted cross-sectional analyses using DNA samples and stress-exposure data from a longitudinal Swedish population-based study (*N* = 2,915). Specifically, we genotyped rs2023239, a functional C/T single nucleotide polymorphism in *CNR1*, previously reported to be associated with CNR1 binding in the brain, subjective reward following alcohol intake, and alcohol cue-elicited brain activation. Our two outcomes of interest were (i) problematic alcohol use based on the Alcohol Use Disorders Identification Test (AUDIT), and (ii) personality trait scores based on the Five Factor Model. We found no baseline association between rs2023239 and problematic alcohol use or personality traits. However, there was a clear trend for interaction between rs2023239’s risk allele (C) and stressful life events (SLEs) in both childhood and adulthood, which predicted problematic alcohol use. Although not significant, there was also some indication that the risk allele interacted with child SLEs to increase scores on neuroticism. Our study supports the notion that the ECS can affect alcohol intake behaviors by interacting with life adversities and is—to the best of our knowledge—the first to focus on the interaction between *CNR1* and stressors in both childhood and adulthood in humans. Further studies are warranted to confirm these findings.

## Introduction

Problematic alcohol use is one of the leading risk factors for both deaths and Disability Adjusted Life Years (DALYs) worldwide^1^. Moreover, there is evidence that the alcohol-induced burden will increase during and after the lockdowns imposed by the COVID-19 pandemic^2,3^. Besides the significant negative consequences on both physical and mental health, there is currently a large gap in the treatment of alcohol use disorders^4,5^. Specifically, it has been estimated that up to 80% of individuals affected by alcohol dependence do not receive adequate treatment due to factors related, among others, to ineffective therapies^4–6^. To develop better pharmacotherapies for alcohol use disorders we need to identify suitable biological targets and disentangle the interplay of various risk factors that contribute to problematic alcohol use. Among these risk factors are both genetic liabilities and exposures to stressful life events in childhood and adulthood^7–11^.

Increasing evidence suggests a critical role for the endocannabinoid system in brain reward processes and addiction^12^. The addictive effects of alcohol, in particular, have been found to be mediated by endocannabinoids that signal via the cannabinoid-1 receptor, which is encoded by the *CNR1* (*CB1R*) gene^13^. In addition, brain regions mediating emotional behavior, such as the amygdala, hippocampus, and cortex, contain high densities of CNR1 receptors that are involved in the regulation of stressful responses and stress-stimulated alcohol intake^14,15^. Candidate genetic studies in humans have found associations between variations in the *CNR1* gene and the use of various substances, including alcohol, cannabis, cocaine, and nicotine^16–21^. However, only few of these variations have been linked to functional changes; one of them being rs2023239, a C/T single nucleotide polymorphism. Specifically, risk-allele (C) carriers of rs2023239 have been found to experience greater subjective reward when consuming alcohol, have higher CNR1 binding in the brain, and have increased risk for polysubstance use^22–24^. Risk-allele carriers have also been found to have enhanced alcohol cue-elicited brain activation in the midbrain and prefrontal cortex^24^, as well as enhanced activity in the orbitofrontal cortex, inferior frontal gyrus, and anterior cingulate gyrus, during exposure to cannabis cues^25^. The risk allele has also been found to associate with higher levels of anger-hostility following cannabis use^26^ and to predict lower hippocampal volumes in cannabis users relative to controls^27^.

Nonetheless, genome-wide association studies (GWAS) have failed to identify a significant association between *CNR1* and problematic alcohol use^28^. However, no studies to our knowledge have examined whether this ECS-related “missing heritability” can be explained by unexamined gene-environment interactions. To this end, we utilized a longitudinal population-based study from Sweden to examine whether *CNR1*’s rs2023239 can interact with stressful life events, experienced in either childhood or adulthood, to predict current problematic alcohol use. Moreover, it has been suggested that a combined assessment of genetic and personality factors is beneficial for a better understanding of AUDs^29^. Since *CNR1* has previously been associated with personality traits^30,31^, which in turn have been linked to different substance abuse behaviors^32^, we also tested for associations between rs2023239 and the Five Factor Model (FFM) of human personality. Overall, our data provide novel insights into the crosstalk between the brain’s endocannabinoid system and environmental stressors, which can modulate the risk for problematic alcohol use.

## Materials and methods

### Participants

Our study was conducted using participants from ‘PART’, a prospective cohort study exploring mental health, work status and relations in adult subjects (20–64 years of age) living in Stockholm County, Sweden^33^. PART included three waves of data collection: wave I (1998–2000; response rate: 53%, *N* = 10,443), wave II (2001–2003; response rate: 84%, *N* = 8613) and wave III (2010–2011; response rate: 66%, *N* = 5650)^34,35^. Data used for the present study included biological samples and information derived from self-administered questionnaires in the different waves covering demographics, child and adult stressful life events, substance use, mental health, and personality traits (as described below). The PART study follows the Code of Ethics of the World Medical Association's (WMA) Declaration of Helsinki, and it has been approved by the ethical review board at Karolinska Institutet (nr. 96-260 and 97-313 for questionnaire data, nr. 2004–528/3 for DNA data, and nr. 2009/880-31 for PART wave III). All described methods were carried out in accordance with relevant guidelines and regulations, and all participants provided informed consent.

### Psychiatric measures

To examine problematic alcohol use, we used the Alcohol Use Disorders Identification Test (AUDIT), which shows good performance in the general population and has also been validated for use in the PART cohort^36–38^. AUDIT scores range from 0 to 40 and problematic alcohol use was operationalized as AUDIT ≥ 8. This cut-off was chosen in order to capture the full range of potentially health-harming drinking behavior, with the score range from 8 to 40 encompassing suspected hazardous and harmful drinking, as well as alcohol dependence^37,39^. AUDIT scores were assessed in PART wave I, unless otherwise indicated, and refer to the respondents’ drinking habits over the past 12 months from the time of filling in the questionnaire. Since problematic alcohol use is highly comorbid with anxiety and depression^40^, we adjusted for these outcomes (assessed in PART waves I and II) in our gene-environment analyses. The presence of a depressive disorder (major depression, mixed anxiety depression or dysthymia according to DSM-IV) was evaluated using the Major Depression Inventory (MDI) coupled with questions on disability due to psychological symptoms^41,42^, as previously described^43^. The assessment questions refer to a period of 14 days prior to filling in the questionnaire. The presence of an anxiety disorder (DSM-IV) was evaluated using the Sheehan Patient Rated Anxiety Scale^44^, the phobia/avoidance part of an instrument developed by Marks and Mathews^45^, and screening questions suggested by the Swedish Psychiatric Association and the Swedish Institute for Health Services Development for obsessive–compulsive disorders, as previously described^46^. Anxiety related questions were phrased in a general manner (“anxiety in certain places or situations”), while the questions for obsessive–compulsive disorders relate to the past 30 days. Depression or anxiety in any of waves I or II were recorded as ‘yes/no’ because of considerable comorbidity and recurrence rate over short time intervals.

### Stressful life events (SLEs)

We examined the impact of self-reported SLEs, assessed in PART wave I, which occurred in childhood or adulthood, as previously described^47–49^. In brief, the following SLEs were considered in childhood (i.e., before the age of 18): (i) loss of at least one parent, (ii) parental divorce, (iii) severe financial difficulties, and (iv) severe family frictions. SLEs in childhood were treated both as a binary variable (cut-off ≥ 1 adversity) and as a 3-level categorical variable (0/1/2–4 adversities). In adulthood, the SLEs considered were reported to have occurred within 12 months prior to filling in the questionnaire and involved data on 28 stressful items including separation, interpersonal conflicts, serious problems/conflicts at work, abortion, severe illness/death of a loved one, and family member victimization, as previously described^47^. Adult SLEs were treated both as a binary variable (cut-off 2 or 3 events) and as a 3-level categorical variable (0–1/2/ ≥ 3 events).

### Personality traits

Personality traits were assessed in PART wave III using a Swedish translation of the Schafer's Five Factor Model (FFM) rating scale^50,51^. Each FFM trait (i.e., conscientiousness, openness, extraversion, agreeableness, and neuroticism) was evaluated using six items with scores of 1–9 (low–high), as previously described^47,48^. The resulting total score for each item (i.e., 6–54) was standardised using z-scores.

### DNA collection and genotyping

DNA samples were obtained using self-administered saliva collection kits (Oragene DNA sample collection kit; DNA Genotek Inc., Canada) from a subset of participants (*N* = 3018) who responded in waves I and II of PART, as previously described^52,53^. For the purposes of the present study, genotyping of rs2023239 was performed in *N* = 2915 individuals using a TaqMan SNP genotyping assay on an ABI 7900 HT instrument (Thermo Fisher Scientific, Waltham, MA, USA). *N* = 88 genotyping reactions (3.02%) could not be clearly allocated to a specific genotype and were excluded in subsequent analyses. To assess quality of genotyping, we tested for deviation from the Hardy–Weinberg equilibrium among observations without a psychiatric diagnosis of depression or anxiety.

### Statistical analyses

Analyses were mostly performed cross-sectionally. Differences in socio-demographic characteristics across categories of problematic alcohol use were assessed using two-sample t-tests or chi-squared tests. Unless otherwise noted, genotype information from rs2023239 was coded as a binary variable (TT versus CT + CC), since the presence of one C allele has been found to have an effect^24^. To examine gene-environment interactions, we first tested for crude individual associations of the exposures with the main outcome (i.e., problematic alcohol use in PART wave I) using logistic regression. We also examined whether the effect estimates of SLEs vary across genotype strata both in univariate analyses and after adjusting for age (in PART wave I), sex, and having received a diagnosis of anxiety or depression (in PART wave I or II). In analyses where the main exposure was adult SLEs, we also adjusted for SLEs in childhood, to estimate the effects for adult SLEs irrespective of having experienced child stress. Since only *N* = 808 participants had experienced 0 SLEs over the past 12 months, we considered it unjustifiable to have 0 adult SLEs as reference category and, to this end, we combined 0 and 1 adult SLEs into one category, which then amounted to about 58% of the sample. Two methodological assessments of gene-environment interactions as departure from additivity were performed, both contrasting the sum of the individual effect estimates with the effect estimate of the doubly exposed group. Specifically, we tested for binary*binary interaction by calculating the relative excess risk due to interaction (RERI)^54^ on problematic alcohol use. To increase statistical power, additive interaction was also evaluated using two-way fixed effects ANOVA models on AUDIT score as a continuous variable. Interaction analyses were adjusted for age, sex, and a diagnosis of anxiety or depression. We tested for normality across all combinations of exposure categories and homogeneity of variances prior to running these ANOVA models. Furthermore, we conducted secondary analyses using sex-specific AUDIT cut-offs. To this end, a lower AUDIT cut-off (≥ 6) was tested for females according to previous studies on problematic or hazardous alcohol use^55–57^. We also conducted analyses combining interaction between rs2023239 and adult SLEs from PART wave I with problematic alcohol use assessed in PART wave II. To explore associations between genotype and personality traits, in the presence or absence of SLEs, two-way ANOVA models were used. For associations between personality traits (assessed only in PART wave III) and problematic alcohol use, logistic regressions were performed with AUDIT scores (assessed in PART wave III), and with adjustment for age and sex. All statistical analyses were performed using Stata 15.1 (StataCorp LLC, TX, USA) with alpha (α) set at ≤ 0.05, not corrected for multiple comparisons.

## Results

### Participant and genotype characteristics

The socio-demographic characteristics of the study participants with information on alcohol use and psychiatric diagnoses (*N* = 2857) are displayed in Table 1 and show that being male, having experienced child or adult SLEs, and having anxiety or depression, were significantly associated with problematic alcohol use (AUDIT ≥ 8) at a level of *p* ≤ 0.01. There was also evidence for between-group differences in problematic alcohol use across education levels (primary/secondary/tertiary). However, since socio-demographic characteristics were not the designated focus of this study and are mainly presented to provide an understanding of the study population’s setup, we will not explore these associations further. Nonetheless, we adjusted for those factors showing the strongest association with problematic alcohol use (sex, age, diagnosis of anxiety or depression) in order to approximate a direct effect of the gene-environment interaction on problematic alcohol use, as well as to control for potential confounding. The genotyping results of rs2023239 did not deviate from Hardy–Weinberg equilibrium and the observed frequencies were *N* = 1983 TT carriers (70.15%), *N* = 787 CT carriers (27.84%) and *N* = 57 CC carriers (2.02%), which is in line with the aggregate allele frequencies reported for the European population by dbGaP (T = 0.83319, C = 0.16681, sample size = 20,502; Alpha Allele Frequency release version: 20201027095038). Supplemental Fig. 1 shows the median AUDIT score, interquartile range, and outliers across genotype categories.

**Table 1:** Socio-demographic characteristics of the study population^†^ and corresponding significance tests of between-group differences across AUDIT score categories.

Characteristic	Category	AUDIT < 8	AUDIT ≥ 8	Total number of observations per comparison (*n*)
Age**; mean (SD)		45.0 (11.9)	40.5 (13.2)	n/a
Sex**; *n* (%)	Male	1010 (39.3)	172 (60.4)	
	Female	1562 (60.7)	113 (39.6)	2857
Education*; *n* (%)	Primary	406 (15.9)	43 (15.1)	
	Secondary	937 (36.6)	125 (44.0)	
	Tertiary	1218 (47.6)	116 (40.8)	2845
Child SLE**; *n* (%)	0	1866 (76.23)	181 (66.3)	
	≥ 1	582 (23.8)	92 (33.7)	2721
Adult SLE**; *n* (%)	0 or 1	1,495 (59.1)	134 (47.5)	
	2	509 (20.1)	64 (22.7)	
	≥ 3	524 (20.7)	84 (29.8)	2810
Anxiety/depression diagnosis**; *n* (%)	No diagnosis	2077 (80.8)	167 (58.8)	
	Diagnosis	492 (19.2)	117 (41.2)	2853
Total		2572 (90.02)	285 (9.98)	2857

*SLE* Stressful life event; *p ≤ 0.05, **p ≤ 0.01.

^†^*N* = 58 observations had missing values for AUDIT score and were therefore excluded. In the comparison of AUDIT score across education categories, an additional 12 observations were excluded due to missing values for the education variable. Similarly, an additional *N* = 136 observations for child SLE, *N* = 47 observations for adult SLE, and *N* = 4 for diagnosis of anxiety or depression were excluded in the respective comparisons.

### No cross-sectional association between rs2023239 and problematic alcohol use

In the univariate logistic regression, there was no statistically significant association between rs2023239 genotype and problematic alcohol use; the odds ratio (OR) for the risk-allele containing genotypes (CC + CT) was 1.14 (95% CI: 0.87–1.49, *p* = 0.34; Table S1). Individuals with SLEs (≥ 1) in childhood had 1.6-fold significantly increased odds of problematic alcohol use compared to those without child SLEs (Table S1). Similarly, individuals with ≥ 2 or ≥ 3 SLEs in adulthood had 1.6-fold significantly increased odds of problematic use compared to those having experienced fewer adult SLEs (Table S1). When testing for effect heterogeneity of stress exposure across genotype strata for child SLEs (0/ ≥ 1), the OR estimates in the TT and CC groups were not statistically significant in either the crude or the adjusted models, but reached significance in the CT group, with an OR of 2.2 in the crude model and an OR of 2.0 in the adjusted model (Table S2). A similar tendency was also observed for adult SLEs (Tables S3–S5).

### Evidence for interaction between rs2023239 and SLEs on risk for problematic alcohol use

We evaluated the interaction of rs2023239 with (i) child SLEs and (ii) adult SLEs on problematic alcohol use, i.e., AUDIT ≥ 8. (i) There was no evidence for departure from additivity due to a binary*binary interaction between the risk-allele containing genotypes and child SLEs (0/ ≥ 1) in the crude analysis. However, there was evidence for departure from additivity on a relative scale (i.e., RERI estimate) when adjusting for age, sex and a diagnosis of anxiety or depression (RERI = 0.83, 95% CI: 0.00–1.67, *p* = 0.05; Table 2). (ii) There was similar evidence for interaction between the risk-allele containing genotypes and adult SLEs (≥ 2) with a crude RERI estimate of 0.77 (95% CI: 0.07–1.47, *p* = 0.03; Table S6). However, the estimated *p*-value increased (> 0.05) following adjustment for age, sex, child SLEs and a diagnosis of anxiety or depression (Table S6). Moreover, there was no evidence for interaction when testing a cut-off of ≥ 3 adult SLEs (Table S6). We also performed sex-stratified RERI analyses for both child SLEs and adult SLEs, which showed evidence for positive additive interaction between child SLEs and rs2023239 only among women. None of the estimates involving adult SLEs provided evidence for departure from additivity (Table S7). Furthermore, we explored interactions between rs2023239 and adult SLEs in PART wave I and problematic alcohol use in PART wave II, to ensure a more certain time sequence of exposure and outcome. Adjusted RERI estimates were similar to those using outcome information from PART wave I, i.e., the crude estimate for adult SLE (≥ 2) was associated with the lowest *p*-value, with all of the other estimates having *p*-values distinctly higher than 0.05 (Table S8).

**Table 2:** Additive interaction between CNR1’s rs2023239 genotype and child SLEs on the odds of problematic alcohol use (i.e., AUDIT score ≥ 8).

	< 1 SLEs	OR (95% CI)	≥ 1 SLEs	OR (95% CI)	OR (95% CI) for SLE within strata of genotype
	N case/control		N case/control		
TT	126/1257	1	53/406	1.08 (0.76, 1.55) *p* = 0.66	1.1 (0.77, 1.58) *p* = 0.61
CC and CT	51/557	0.9 (0.63, 1.27) *p* = 0.53	33 / 160	1.81 (1.16, 2.83) *p* ≤ 0.01	1.99 (1.18, 3.34) *p* ≤ 0.01
ORs (95% CI) for genotype within strata of SLEs		0.89 (0.63, 1.27) *p* = 0.53		1.67 (1.01, 2.74) *p* = 0.04	

Case/control refers to problematic alcohol use (yes/no).

*OR* Odds Ratios, *CI* Confidence Interval, *SLE* Stressful life event.

Estimates adjusted for age, sex, and diagnosis of anxiety or depression.

Crude RERI estimate 0.84 (95% CI: − 0.05–1.74; *p* = 0.07).

Adjusted RERI estimate 0.83 (95% CI: 0.0–1.67; *p* = 0.05), calculated with values from table above: 1.81 − 0.9 − 1.08 + 1 = 0.83.

**Figure 1:**
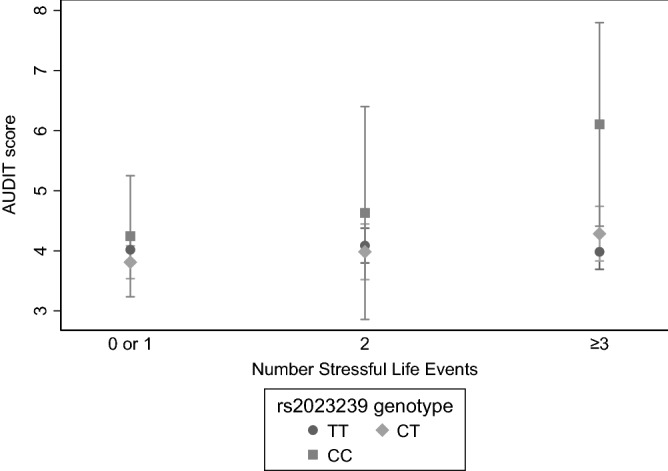
Testing for interaction between rs2023239’s C-allele and number of adult stressful life events (SLEs) on predicted AUDIT scores in PART I. Mean predicted continuous AUDIT score by rs2023239 genotype (TT, CT, CC) and three-level categorical adult SLEs (0/1, 2 or ≥ 3), adjusted for age, sex, child SLEs (≥ 1) and a diagnosis of anxiety or depression. Results from the two-way ANOVA suggested a tendency toward effect heterogeneity for SLE exposure, with risk-allele carriers experiencing a sharper increase in AUDIT scores across SLE categories, although the interaction term did not reach statistical significance (adjusted *p* = 0.23). Error bars represent 95% confidence intervals.

Next, we also evaluated interactions by using the AUDIT score as a continuous outcome. Results from the two-way ANOVA using three-level categorical variables for both genotype and adult SLEs suggested a tendency toward effect heterogeneity for SLE exposure, with risk-allele carriers experiencing a sharper increase in AUDIT scores across SLE categories, although the interaction term had a *p*-value of > 0.05 (Fig. 1). For adult SLEs (0–2 versus ≥ 3), the interaction term was ≤ 0.05 in the crude model (*p* = 0.04, adjusted *p* = 0.06; data not shown). This was also the case when considering an adult SLE cut-off of ≥ 2 events (i.e., 0–1 versus ≥ 2 SLEs: crude *p* = 0.03, adjusted *p* = 0.14; data not shown). Corresponding results for child SLEs are omitted since there were no observations with the joint exposure of CC genotype and having experienced 2–4 SLEs in childhood. However, when running the ANOVA with binary exposure variables, the *p*-values for the interaction terms for genotype (TT versus CT + CC) and child SLEs (0 versus ≥ 1) were low in both the crude and adjusted ANOVA model (*p* ≤ 0.01; data not shown).

### Associations between rs2023239 and personality traits

To examine associations between rs2023239, AUDIT scores and the five FFM personality traits (i.e., extraversion, neuroticism, conscientiousness, openness, and agreeableness), individuals that responded in PART wave III were used (see also Materials and Methods), which constituted a subset of the genotyped individuals (*N* = 2222; Table S9). The distribution of trait scores by genotype indicated a trend for association between the risk (C) allele of rs2023239 and higher extraversion (Fig. S1), as well as lower agreeableness and neuroticism (Fig. S3). However, in both crude and adjusted (for age and sex) regression models, no statistically significant associations could be found (data not shown). In addition, there was some evidence for a differential impact of child SLEs on FFM scores across rs2023239 genotypes (Figs. S4–S6). For instance, there was a tendency towards increased neuroticism in individuals who had experienced child SLEs, and this tendency was strongest among C-allele homozygotes, but the interaction terms in the regression model did not reach statistical significance (Fig. S5; interaction child SLE#CT, *p* = 0.78; child SLE#CC, *p* = 0.09). Finally, we also examined baseline associations between FFM traits and the risk for problematic alcohol use. Higher neuroticism was associated with increased odds of exhibiting problematic alcohol use (adjusted OR = 1.4, 95% CI: 1.22–1.6, *p* ≤ 0.01; Table S10). Moreover, higher conscientiousness and higher agreeableness were associated with lower odds of exhibiting problematic alcohol use (conscientiousness: adjusted OR = 0.75, 95% CI: 0.66–0.85, *p* ≤ 0.01; agreeableness: adjusted OR = 0.8, 95% CI: 0.7–0.93, *p* ≤ 0.01; Table S8).

## Discussion

Preclinical studies have consistently supported a role for the endocannabinoid system (ECS) in affecting reward- and substance use-related behaviors^58–66^. With regard to alcohol, in particular, genetic deletion of the cannabinoid receptor-1 gene (*Cnr1*) or treatment with rimonabant (a CNR1 inverse agonist) have been found to attenuate a number of alcohol-mediated outcomes and behaviors, including voluntary drinking, operant alcohol self-administration, reinstatement of alcohol-seeking behaviors, alcohol-induced dopamine release in the nucleus accumbens, conditioned place preference and behavioral sensitization^67–70^. These addiction-related studies have suggested that an enhanced endocannabinoid tone in the mesolimbic pathway facilitates the rewarding effects of commonly abused substances^69,71,72^.

Besides its role in brain reward processes, the ECS is also implicated in habituation to repeated stress and functions as an important modulatory system throughout the corticolimbic circuit, buffering against the negative effects of stress^73–78^. Importantly, preclinical studies have also suggested a crosstalk between the ECS and environmental stress, which impacts reward-related behaviors, e.g., demonstrated by the lack of stress-induced alcohol preference in *Cnr1* knockout mice; a behavior which is normally observed in wild-type animals^15^. It has also been found that maternal separation (MS) induces changes in endocannabinoid levels and CNR1 expression, which might underlie the MS-induced increase in alcohol intake^79,80^. Significant interactions between restraint stress and alcohol exposure on the expression of major endocannabinoids, such as 2-arachidonoyl glycerol (2-AG), have also been observed in the rat amygdala^81^. Moreover, pharmacological studies have found that the enhanced anxiety-like behavior and alcohol consumption observed in alcohol-dependent rats, is attenuated by 2-AG hydrolysis inhibitors^82^. Finally, in human studies, the risk (C) allele of rs2023239, including higher levels of early life stress and current perceived stress, have been found to be overrepresented in cannabis users^83^.

However, direct human evidence for gene-environment interactions that involve the ECS and modulate substance use behaviors has been lacking. To this end, we utilized the presence of a functional genetic variant in the human *CNR1* gene, i.e., rs2023239, to study the putative role of the ECS in affecting alcohol intake levels. Risk-allele (C) carriers of rs2023239 have previously been found to have higher CNR1 binding in the prefrontal cortex, to experience enhanced subjective reward following alcohol intake, and to show greater alcohol cue-elicited brain activation both in the midbrain and the prefrontal cortex^23,24^. In our utilized population-based cohort from Sweden, and in line with the latest GWAS^28^, we found no baseline association between rs2023239 and problematic alcohol use, defined as AUDIT scores ≥ 8^37,39^. By contrast, however, we found evidence for a gene-environment interaction that impacted the risk for problematic alcohol use.

Specifically, there was a clear trend in our data suggesting that risk (C) allele carriers have an increased risk for problematic alcohol use when exposed to stressful life events (SLEs) in either childhood or adulthood. For instance, compared to the non-risk homozygous (TT) group, CT-carriers had an approximately two-fold increase in odds of exhibiting problematic alcohol use following SLE exposure. However, estimates from CC-carriers were not as informative due to wide confidence intervals resulting from a small number of observations with this genotype and SLE exposure. Nonetheless, it is reassuring that both child and adult SLEs showed the same pattern across genotype strata, and that the C-allele has consistently been reported as the risk allele in previous substance use-related studies^22–27^. Our findings are further supported by the statistically significant RERI estimate for child SLEs, pointing to a synergistic effect between the C-allele and SLE exposure that were statistically significant after adjustment for putative confounders. Although RERI estimates for adult SLEs did not consistently reach statistical significance, they also pointed to the same direction. Furthermore, when using the continuous AUDIT score as the outcome and, thereby, increasing power to detect additive interaction between genotype and SLEs, binary*binary interaction terms also showed statistical significance.

Collectively, our analyses provide the first evidence, to our knowledge, for a synergistic interaction between the risk allele of rs2023239 and SLEs, which increases the risk for developing problematic alcohol use. Although there is no single causal pathway leading from SLEs to problematic drinking, it is known that perceived stress can predict the development of a harmful relationship to alcohol^10,84–87^. Since C-allele carriers of rs2023239 have been found to have greater subjective reward to alcohol and greater alcohol cue-elicited brain activation^24^, it can be hypothesized that the C-allele constitutes a risk factor in terms of enhanced positive responses to alcohol which, in combination with alcohol’s acute anxiolytic properties^88^, may feed into the maintenance of problematic alcohol use. Another possibility, which warrants future examination, is that the C-allele of rs2023239 contributes to enhanced stress vulnerability that increases the risk to turn to alcohol for self-medication purposes^88^.

The stress vulnerability of an individual is associated with both external and internal factors, such as socioeconomic status and personality traits^89–91^. Personality traits, in particular, have been suggested to play a critical role in the development of substance use disorders, with Five Factor Model (FFM) traits, such as higher neuroticism and lower conscientiousness, typically serving as substance use predictors^92–94^. In line with these previous studies, our FFM-related analyses in the present Swedish cohort found higher neuroticism and lower conscientiousness to be associated with problematic alcohol use. Previous studies have found significant associations between genetic variations in *CNR1* and FFM traits, although not with rs2023239^30,31^. Accordingly, our exploratory analyses of rs2023239 and FFM trait expression, in the presence or absence of SLEs, also remain inconclusive. For example, although there was some indication that homozygosity for the risk (C) allele can interact with child SLEs to increase neuroticism (which would again be in line with the C-allele conferring risk for problematic alcohol use), this interaction did not reach statistical significance and warrants investigation in other cohorts.

Our study had a number of limitations that need to be acknowledged, with the first being the relatively small sample size that also precluded the use of a genome-environment-wide interaction study (GEWIS) approach. For instance, the largest GEWIS of alcohol misuse, to date, was conducted for trauma exposure using a discovery cohort of *N* = 16,361, and a replication cohort of *N* = 8084, and succeeded in uncovering only one genome-wide significant hit that survived replication^95^. In our study, there was also a very small number of observations with extreme AUDIT scores in the homozygote risk (CC) group, which pulled the effect estimates in the analyses of binary*binary interactions upwards. The C-allele has a frequency of approx. 16% in the European population and is relatively uncommon in homozygosity, e.g., only 2% of our participants were homozygotes for the risk allele. Previous GWAS have found nominally significant associations between the C-allele and addiction-related outcomes, including the number of unsuccessful stop-smoking attempts and the frequency of feeling guilt or remorse after drinking alcohol in the last year^96^. Thus, the lack of significant main effects for rs2023239 in both our and previous studies^37,39^, could still be the outcome of underpowered analyses, and with future larger cohorts being able to uncover a significant association with problematic alcohol use. However, as GWAS cohorts of alcohol use get larger and larger, and since life adversities are known to be overrepresented in psychiatric cohorts, any significant main effects eventually achieved could still reflect the presence of underlying gene-environment interactions that warrant investigation using sensitivity analyses. In our study, we also did not adjust for multiple comparisons in any of the analyses, which weakens the strength of evidence we are able to present. However, since (i) the effect estimates for SLEs in the CT group had relatively low *p*-values, without any observable effect in the TT group, and (ii) even if not statistically significant, all estimates from the ANOVA, RERI and stratified analyses pointed in the same direction, we remain confident in reporting evidence for a synergistic interaction between the C allele of rs2023239 and SLE exposure in both childhood and adulthood. Moreover, while adjustment for certain co-morbidities, such as anxiety and depression, was possible in our study, we did not have data to adjust for all psychiatric disorders that might be of relevance for substance use studies. However, we consider this to be a minor limitation, since anxiety and depression are the most prevalent psychiatric disorders in comparable settings^97^.

A strength of this study was the two-tier approach to test for interaction, with both univariate and multivariate stratified analyses as the first step, and analyses of joint exposure effects as the second step. Moreover, no major entry sources of bias could be identified, and while most of the data were self-reported, this is unlikely to have led to differential misclassification of either outcome or exposures. The AUDIT score, in particular, is a widely utilized tool, which has been validated for use in our investigated cohort^36,37^. However, in our study we focused mainly on problematic alcohol use defined as AUDIT ≥ 8, and for future larger studies it could be advisable to test additional AUDIT thresholds. Furthermore, a more detailed investigation of sex-specific rs2023239—stress interaction patterns may be of interest, given that our sex-stratified secondary analyses suggested slight differences between men and women. Future studies could also benefit from taking a longitudinal approach to the development of problematic alcohol use and the role of gene-environment interaction in alcohol use trajectories by considering alcohol use in childhood and adolescence, as well as from a clearer time sequence between adult exposures and outcome. Furthermore, it would strengthen the evidence presented in this paper to replicate the gene-environment interactions under study here in relation to other substance use disorders.

## Conclusion

Although further studies are warranted to confirm the present findings, our study provides continued support for the involvement of the endocannabinoid system in affecting substance use behaviors. Moreover, it suggests that pharmacological manipulation of endocannabinoid signaling, e.g., with the use of cannabinoid receptor allosteric modulators that circumvent the negative neuropsychiatric side effects of antagonists or inverse agonists, continues to hold promise as an effective intervention for treating problematic alcohol use^98,99^.

## Supplementary Information

Supplementary Information.

## References

[1] Collaborators GBDA (2018). Alcohol use and burden for 195 countries and territories, 1990–2016: a systematic analysis for the global burden of disease study 2016. Lancet.

[2] Mallet J, Dubertret C, Le Strat Y (2021). Addictions in the COVID-19 era: Current evidence, future perspectives a comprehensive review. Prog. Neuropsychopharmacol. Biol. Psychiatry.

[3] Pollard MS, Tucker JS, Green HD (2020). Changes in Adult Alcohol Use and Consequences During the COVID-19 Pandemic in the US. JAMA Netw. Open.

[4] Jerlhag E (2019). Gut-brain axis and addictive disorders: A review with focus on alcohol and drugs of abuse. Pharmacol. Ther. (Oxf.).

[5] Heilig M, Augier E, Pfarr S, Sommer WH (2019). Developing neuroscience-based treatments for alcohol addiction: A matter of choice?. Transl. Psychiatry.

[6] Rehm J (2015). Alcohol dependence and treatment utilization in Europe – a representative cross-sectional study in primary care. BMC Fam. Pract..

[7] Lee RD, Chen J (2017). Adverse childhood experiences, mental health, and excessive alcohol use: Examination of race/ethnicity and sex differences. Child Abuse Negl..

[8] Hughes K (2017). The effect of multiple adverse childhood experiences on health: a systematic review and meta-analysis. Lancet Public Health.

[9] Diggs ON, Neppl TK (2018). The Influence of economic pressure on emerging adult binge drinking: testing the family stress model over time. J. Youth Adolesc..

[10] Peltier MR (2019). Sex differences in stress-related alcohol use. Neurobiol. Stress.

[11] Cohen S, Murphy MLM, Ten Prather AA (2019). Surprising facts about stressful life events and disease risk. Annu. Rev. Psychol..

[12] Parsons LH, Hurd YL (2015). Endocannabinoid signalling in reward and addiction. Nat. Rev. Neurosci..

[13] Kunos G (2020). Interactions between alcohol and the endocannabinoid system. Alcohol Clin. Exp. Res..

[14] Viveros MP, Marco EM, File SE (2005). Endocannabinoid system and stress and anxiety responses. Pharmacol Biochem Behav.

[15] Racz I (2003). A critical role for the cannabinoid CB1 receptors in alcohol dependence and stress-stimulated ethanol drinking. J. Neurosci..

[16] Zuo L, Kranzler HR, Luo X, Covault J, Gelernter J (2007). CNR1 variation modulates risk for drug and alcohol dependence. Biol. Psychiatry.

[17] Hopfer CJ (2006). Cannabis receptor haplotype associated with fewer cannabis dependence symptoms in adolescents. Am. J. Med. Genet. B Neuropsychiatr. Genet..

[18] Schmidt LG (2002). Association of a CB1 cannabinoid receptor gene (CNR1) polymorphism with severe alcohol dependence. Drug Alcohol Depend.

[19] Comings DE (1997). Cannabinoid receptor gene (CNR1): association with iv drug use. Mol. Psychiatry.

[20] Ballon N (2006). (AAT)n repeat in the cannabinoid receptor gene (CNR1): association with cocaine addiction in an African-Caribbean population. Pharmacogn. J..

[21] Chen X (2008). Cannabinoid receptor 1 gene association with nicotine dependence. Arch. Gen. Psychiatry.

[22] Zhang PW (2004). Human cannabinoid receptor 1: 5' exons, candidate regulatory regions, polymorphisms, haplotypes and association with polysubstance abuse. Mol. Psychiatry.

[23] Hirvonen J (2013). Reduced cannabinoid CB1 receptor binding in alcohol dependence measured with positron emission tomography. Mol. Psychiatry.

[24] Hutchison KE (2008). The incentive salience of alcohol: translating the effects of genetic variant in CNR1. Arch. Gen. Psychiatry.

[25] Filbey FM, Schacht JP, Myers US, Chavez RS, Hutchison KE (2010). Individual and additive effects of the CNR1 and FAAH genes on brain response to marijuana cues. Neuropsychopharmacology.

[26] Palmer RHC, McGeary JE, Knopik VS, Bidwell LC, Metrik JM (2019). CNR1 and FAAH variation and affective states induced by marijuana smoking. Am. J. Drug Alcohol Abuse.

[27] Schacht JP, Hutchison KE, Filbey FM (2012). Associations between cannabinoid receptor-1 (CNR1) variation and hippocampus and amygdala volumes in heavy cannabis users. Neuropsychopharmacology.

[28] Zhou H (2020). Genome-wide meta-analysis of problematic alcohol use in 435,563 individuals yields insights into biology and relationships with other traits. Nat. Neurosci..

[29] Oreland L (2018). Personality as an intermediate phenotype for genetic dissection of alcohol use disorder. J. Neural Transm. (Vienna).

[30] Yao Y (2018). Detection of significant association between variants in cannabinoid receptor 1 gene (CNR1) and personality in african-american population. Front Genet.

[31] Juhasz G (2009). CNR1 gene is associated with high neuroticism and low agreeableness and interacts with recent negative life events to predict current depressive symptoms. Neuropsychopharmacology.

[32] Kotov R, Gamez W, Schmidt F, Watson D (2010). Linking, "big" personality traits to anxiety, depressive, and substance use disorders: a meta-analysis. Psychol. Bull.

[33] Hällström, T., Damström Thakker, K., Forsell, Y., Lundberg, I. & Tinghög, P. *The Part Study. A Population Based Study of Mental Health in the Stockholm County: Study Design. Phase l (1998–2000)*, <https://pdfs.semanticscholar.org/3f5a/fa1f8da8011e3c74cbe57cb56321202b9d38.pdf?_ga=2.264430607.1661230138.1568310269-2047038646.1567702029> (2003).

[34] Bergman P, Ahlberg G, Forsell Y, Lundberg I (2010). Non-participation in the second wave of the PART study on mental disorder and its effects on risk estimates. Int. J. Soc. Psychiatry.

[35] Lundberg I, Thakker KD, Hallstrom T, Forsell Y (2005). Determinants of non-participation, and the effects of non-participation on potential cause-effect relationships, in the PART study on mental disorders. Soc. Psychiatry Psychiatr. Epidemiol..

[36] Lundin A, Hallgren M, Balliu N, Forsell Y (2015). The use of alcohol use disorders identification test (AUDIT) in detecting alcohol use disorder and risk drinking in the general population: validation of AUDIT using schedules for clinical assessment in neuropsychiatry. Alcohol. Clin. Exp. Res..

[37] Babor, T. F., Higgins-Biddle, J. C., Saunders, J. B. & Monteiro, M. G. AUDIT: The Alcohol Use Disorders Identification Test. (World Health Organization, 2001).

[38] Moehring A (2019). Diagnostic performance of the Alcohol Use Disorders Identification Test (AUDIT) in detecting DSM-5 alcohol use disorders in the General population. Drug Alcohol Depend.

[39] Saunders JB, Degenhardt L, Reed GM, Poznyak V (2019). Alcohol use disorders in ICD-11: past, present, and future. Alcohol Clin. Exp. Res..

[40] Boschloo L (2011). Comorbidity and risk indicators for alcohol use disorders among persons with anxiety and/or depressive disorders: findings from the Netherlands study of depression and anxiety (NESDA). J. Affect. Disord..

[41] Forsell Y (2005). The major depression inventory versus schedules for clinical assessment in neuropsychiatry in a population sample. Soc. Psychiatry Psychiatr. Epidemiol..

[42] Olsen LR, Jensen DV, Noerholm V, Martiny K, Bech P (2003). The internal and external validity of the major depression inventory in measuring severity of depressive states. Psychol. Med..

[43] Rayman JB (2020). Single-nucleotide polymorphism in the human TIA1 gene interacts with stressful life events to predict the development of pathological anxiety symptoms in a Swedish population. J. Affect. Disord..

[44] Sheehan, D. V. *The Anxiety Disease*. (Charles Scribner's Sons, 1983).

[45] Marks IM, Mathews AM (1979). Brief standard self-rating for phobic patients. Behav. Res. Ther..

[46] Wallerblad A, Moller J, Forsell Y (2012). Care-seeking pattern among persons with depression and anxiety: a population-based study in Sweden. Int. J. Family Med..

[47] Melas PA, Guban P, Rahman MS, Lavebratt C, Forsell Y (2018). Neuropeptide Y, stressful life events and personality trait conscientiousness: Preliminary associations from a Swedish longitudinal study. Psychiatry Res..

[48] Rahman MS (2017). The serotonin transporter promoter variant (5-HTTLPR) and childhood adversity are associated with the personality trait openness to experience. Psychiatry Res..

[49] Liu JJ, Lou F, Lavebratt C, Forsell Y (2015). Impact of childhood adversity and vasopressin receptor 1a variation on social interaction in adulthood: a cross-sectional study. PLoS ONE.

[50] Shafer AB (1999). Brief bipolar markers for the five factor model of personality. Psychol. Rep..

[51] Hochwalder J (2006). A psychometric assessment of a Swedish translation of Shafer's personality scale. Scand. J. Psychol..

[52] Melas PA (2010). Examining the public refusal to consent to DNA biobanking: empirical data from a Swedish population-based study. J. Med. Ethics.

[53] Sjoholm LK, Melas PA, Forsell Y, Lavebratt C (2009). PreproNPY Pro7 protects against depression despite exposure to environmental risk factors. J Affect Disord.

[54] Knol MJ, VanderWeele TJ (2012). Recommendations for presenting analyses of effect modification and interaction. Int. J. Epidemiol..

[55] Aalto M, Tuunanen M, Sillanaukee P, Seppa K (2006). Effectiveness of structured questionnaires for screening heavy drinking in middle-aged women. Alcohol Clin. Exp. Res..

[56] Lundin A, Hallgren M, Balliu N, Forsell Y (2015). The use of alcohol use disorders identification test (AUDIT) in detecting alcohol use disorder and risk drinking in the general population: validation of AUDIT using schedules for clinical assessment in neuropsychiatry. Alcohol Clin. Exp. Res..

[57] Canfield M, Chandler V, Foster JH (2020). Home drinking in women over 30 years of age. Findings from an internet survey in England. J. Subst. Use.

[58] Scherma M (2020). Cannabinoid exposure in rat adolescence reprograms the initial behavioral, molecular, and epigenetic response to cocaine. Proc. Natl. Acad. Sci. U S A.

[59] Melas PA (2018). Cannabinoid modulation of eukaryotic initiation factors (eIF2alpha and eIF2B1) and behavioral cross-sensitization to cocaine in adolescent rats. Cell Rep.

[60] Kononoff J (2018). Adolescent cannabinoid exposure induces irritability-like behavior and cocaine cross-sensitization without affecting the escalation of cocaine self-administration in adulthood. Sci. Rep..

[61] Godlewski G (2019). Targeting peripheral CB1 receptors reduces ethanol intake via a gut-brain axis. Cell Metab..

[62] Marcus DJ (2017). Mice expressing a "hyper-sensitive" form of the CB1 cannabinoid receptor (CB1) show modestly enhanced alcohol preference and consumption. PLoS ONE.

[63] Ellgren M, Spano SM, Hurd YL (2007). Adolescent cannabis exposure alters opiate intake and opioid limbic neuronal populations in adult rats. Neuropsychopharmacology.

[64] Caille S, Parsons LH (2006). Cannabinoid modulation of opiate reinforcement through the ventral striatopallidal pathway. Neuropsychopharmacology.

[65] De Vries TJ (2001). A cannabinoid mechanism in relapse to cocaine seeking. Nat Med.

[66] Ledent C (1999). Unresponsiveness to cannabinoids and reduced addictive effects of opiates in CB1 receptor knockout mice. Science.

[67] Hungund BL, Szakall I, Adam A, Basavarajappa BS, Vadasz C (2003). Cannabinoid CB1 receptor knockout mice exhibit markedly reduced voluntary alcohol consumption and lack alcohol-induced dopamine release in the nucleus accumbens. J. Neurochem..

[68] Thanos PK, Dimitrakakis ES, Rice O, Gifford A, Volkow ND (2005). Ethanol self-administration and ethanol conditioned place preference are reduced in mice lacking cannabinoid CB1 receptors. Behav. Brain Res..

[69] Cheer JF (2007). Phasic dopamine release evoked by abused substances requires cannabinoid receptor activation. J. Neurosci..

[70] Houchi H (2005). CB1 receptor knockout mice display reduced ethanol-induced conditioned place preference and increased striatal dopamine D2 receptors. Neuropsychopharmacology.

[71] Orio L, Edwards S, George O, Parsons LH, Koob GF (2009). A role for the endocannabinoid system in the increased motivation for cocaine in extended-access conditions. J. Neurosci..

[72] Bilbao A (2020). Endocannabinoid LTD in accumbal D1 neurons mediates reward-seeking behavior. iScience.

[73] Morena M, Patel S, Bains JS, Hill MN (2016). Neurobiological interactions between stress and the endocannabinoid system. Neuropsychopharmacology.

[74] Beins EC (2021). Cannabinoid receptor 1 signalling modulates stress susceptibility and microglial responses to chronic social defeat stress. Transl. Psychiatry.

[75] Hill MN (2010). Endogenous cannabinoid signaling is essential for stress adaptation. Proc. Natl. Acad. Sci. U S A.

[76] Sumislawski JJ, Ramikie TS, Patel S (2011). Reversible gating of endocannabinoid plasticity in the amygdala by chronic stress: a potential role for monoacylglycerol lipase inhibition in the prevention of stress-induced behavioral adaptation. Neuropsychopharmacology.

[77] Hill MN (2011). Recruitment of prefrontal cortical endocannabinoid signaling by glucocorticoids contributes to termination of the stress response. J. Neurosci..

[78] Rademacher DJ (2008). Effects of acute and repeated restraint stress on endocannabinoid content in the amygdala, ventral striatum, and medial prefrontal cortex in mice. Neuropharmacology.

[79] Romano-Lopez A, Mendez-Diaz M, Ruiz-Contreras AE, Carrisoza R, Prospero-Garcia O (2012). Maternal separation and proclivity for ethanol intake: a potential role of the endocannabinoid system in rats. Neuroscience.

[80] Portero-Tresserra M (2018). Maternal separation increases alcohol-drinking behaviour and reduces endocannabinoid levels in the mouse striatum and prefrontal cortex. Eur. Neuropsychopharmacol..

[81] Sanchez-Marin L (2022). Acute stress and alcohol exposure during adolescence result in an anxious phenotype in adulthood: Role of altered glutamate/endocannabinoid transmission mechanisms. Prog. Neuropsychopharmacol. Biol. Psychiatry.

[82] Serrano A (2018). Deficient endocannabinoid signaling in the central amygdala contributes to alcohol dependence-related anxiety-like behavior and excessive alcohol intake. Neuropsychopharmacology.

[83] Filbey FM, Beaton D, Prashad S (2021). The contributions of the endocannabinoid system and stress on the neural processing of reward stimuli. Prog. Neuropsychopharmacol. Biol. Psychiatry.

[84] Stevenson BL (2019). Within- and between-person associations from mood to alcohol consequences: the mediating role of enhancement and coping drinking motives. J. Abnorm. Psychol..

[85] Temmen CD, Crockett LJ (2019). Relations of stress and drinking motives to young adult alcohol misuse: variations by gender. J. Youth Adolesc..

[86] Becker HC (2017). Influence of stress associated with chronic alcohol exposure on drinking. Neuropharmacology.

[87] McCaul ME, Hutton HE, Stephens MAC, Xu X, Wand GS (2017). Anxiety, anxiety sensitivity, and perceived stress as predictors of recent drinking, alcohol craving, and social stress response in heavy drinkers. Alcohol. Clin. Exp. Res..

[88] Smith JP, Randall CL (2012). Anxiety and alcohol use disorders: comorbidity and treatment considerations. Alcohol Res..

[89] Joseph GG, David MA, Shevaun DN, Susan LE (2004). Socioeconomic status and health: a micro-level analysis of exposure and vulnerability to daily stressors. J. Health Soc. Behav..

[90] Diderichsen F, Hallqvist J, Whitehead M (2019). Differential vulnerability and susceptibility: how to make use of recent development in our understanding of mediation and interaction to tackle health inequalities. Int. J. Epidemiol..

[91] Widiger TA, Oltmanns JR (2017). Neuroticism is a fundamental domain of personality with enormous public health implications. World Psychiatry.

[92] Rogers MM, McKinney C, Asberg K (2018). Substance use predicted by parental maltreatment, gender, and five-factor personality. Personal. Individ. Differ..

[93] Raketic D (2017). Five-factor model personality profiles: the differences between alcohol and opiate addiction among females. Psychiatr. Danub..

[94] Delić M, Kajdiž K, Pregelj P (2017). Association of the five-factor model personality traits and opioid addiction treatment outcome. Psychiatr. Danub..

[95] Polimanti R (2018). A genome-wide gene-by-trauma interaction study of alcohol misuse in two independent cohorts identifies PRKG1 as a risk locus. Mol. Psychiatry.

[96] Watanabe K (2019). A global overview of pleiotropy and genetic architecture in complex traits. Nat. Genet.

[97] Sundquist J, Ohlsson H, Sundquist K, Kendler KS (2017). Common adult psychiatric disorders in Swedish primary care where most mental health patients are treated. BMC Psychiatry.

[98] Gianessi CA (2020). Endocannabinoid contributions to alcohol habits and motivation: relevance to treatment. Addict Biol..

[99] Henderson-Redmond AN, Guindon J, Morgan DJ (2016). Roles for the endocannabinoid system in ethanol-motivated behavior. Prog. Neuropsychopharmacol. Biol. Psychiatry.

