# Supplementary material for: The cannabinoid receptor-1 gene interacts with stressful life events to increase the risk for problematic alcohol use
Source: Sci Rep. 2022 Mar 23;12:4963. doi: 10.1038/s41598-022-08980-w (PMC8941304; doi:10.1038/s41598-022-08980-w)
Supplement: Supplementary file 1 — Supplementary Information. [file 41598_2022_8980_MOESM1_ESM.docx]

**Title**

The cannabinoid receptor-1 gene interacts with stressful life events to increase the risk for problematic alcohol use

**Authors**

Lisa Bornscheuer ^1,4^, Andreas Lundin ^2^, Yvonne Forsell ^2^, Catharina Lavebratt ^3,4^, Philippe A. Melas ^4,5, *^

**Affiliations**

^1^ Department of Public Health Sciences, Stockholm University, 10691 Stockholm, Sweden

^2^ Department of Global Public Health, Karolinska Institutet, 17177 Stockholm, Sweden

^3^ Department of Molecular Medicine and Surgery, Karolinska Institutet, 17176 Stockholm, Sweden

^4^ Center for Molecular Medicine, L8:00, Karolinska University Hospital, 17176 Stockholm, Sweden

^5^ Center for Psychiatry Research, Department of Clinical Neuroscience, Karolinska Institutet & Stockholm Health Care Services, 11364 Stockholm, Sweden

**^*^ Correspondence:** L8:00, Karolinska University Hospital, 17176, Stockholm, Sweden. Tel: +46 70 888 44 05. E-mail address: [Philippe.Melas@ki.se](mailto:Philippe.Melas@ki.se)

**Supplemental Material**

**Supplemental Tables**

**Table S1.** Univariate OR estimates following logistic regression of problematic alcohol use (i.e., AUDIT score ≥8) on rs2023239 and SLEs

| **Exposure** | **OR (95% CI) †** | **p-value** |
| --- | --- | --- |
| **CT&CC** | 1.14 (0.87, 1.49) | 0.34 |
| **Child SLEs (≥1)** | 1.63 (1.25, 2.13) | ≤ 0.01 |
| **Adult SLEs (≥2)** | 1.60 (1.25, 2.05) | ≤ 0.01 |
| **Adult SLEs (≥3)** | 1.62 (1.24, 2.13) | ≤ 0.01 |

OR: Odds Ratios; CI: Confidence Interval; SLEs: Stressful Life Events

**†**Crude models

**Table S2.** Logistic regression of problematic alcohol use (i.e., AUDIT score ≥8) on ≥1 SLEs in childhood stratified by rs2023239

| **rs2023239** | **OR (95% CI) †** | **p-value** | **OR (95% CI) ‡** | **p-value** |
| --- | --- | --- | --- | --- |
| **TT** | 1.30 (0.93, 1.83) | 0.13 | 1.10 (0.77, 1.58) | 0.61 |
| **CT** | 2.20 (1.35, 3.58) | ≤ 0.01 | 2.00 (1.17, 3.41) | ≤ 0.01 |
| **CC** | 3.25 (0.47, 22.71) | 0.24 | 0.98 (0.08, 12.6) | 0.99 |

OR: Odds Ratios; CI: Confidence Interval; SLEs: Stressful Life Events

**†**Crude model; **‡**Model adjusted for age, sex, anxiety, depression

**Table S3.** Logistic regression of problematic alcohol use (i.e., AUDIT score ≥8) on ≥2 SLEs in adulthood stratified by rs2023239

| **rs2023239** | **OR (95% CI) †** | **p-value** | **OR (95% CI) ‡** | **p-value** |
| --- | --- | --- | --- | --- |
| **TT** | 1.27 (0.93, 1.72) | 0.13 | 1.16 (0.83, 1.63) | 0.38 |
| **CT** | 2.33 (1.46, 3.72) | ≤ 0.01 | 1.71 (1.0, 2.94) | 0.05 |
| **CC** | 0.89 (0.14, 5.81) | 0.9 | 0.43 (0.04, 4.39) | 0.48 |

OR: Odds Ratios; CI: Confidence Interval; SLEs: Stressful Life Events

**†**Crude model; **‡**Model adjusted for age, sex, anxiety, depression, child SLEs (0/≥1)

**Table S4.** Logistic regression of problematic alcohol use on ≥3 SLEs in adulthood stratified by rs2023239

| **rs2023239** | **OR (95% CI) †** | **p-value** | **OR (95% CI) ‡** | **p-value** |
| --- | --- | --- | --- | --- |
| **TT** | 1.30 (0.91, 1.85) | 0.14 | 1.11 (0.75, 1.65) | 0.59 |
| **CT** | 2.26 (1.38, 3.68) | ≤ 0.01 | 1.71 (0.96, 3.05) | 0.07 |
| **CC** | 0.77 (0.08, 7.58) | 0.82 | 0.46 (0.03, 7.05) | 0.58 |

OR: Odds Ratios; CI: Confidence Interval; SLEs: Stressful Life Events

**†**Crude model; **‡**Model adjusted for age, sex, anxiety, depression, child SLEs (0/≥1)

**Table S5.** Logistic regression of problematic alcohol use on 0-1/2/≥3 adult SLEs stratified by rs2023239

| **rs2023239** | **Adult SLEs** | **OR (95% CI) †** | **p-value** | **OR (95% CI) ‡** | **p-value** |
| --- | --- | --- | --- | --- | --- |
| **TT** | 2 | 1.17 (0.79, 1.74) | 0.42 | 1.16 (0.77, 1.74) | 0.49 |
|  | ≥3 | 1.36 (0.94, 1.96) | 0.1 | 1.17 (0.77, 1.76) | 0.47 |
| **CT** | 2 | 1.91 (1.06, 3.44) | 0.03 | 1.46 (0.75, 2.83) | 0.26 |
|  | ≥3 | 2.75 (1.61, 4.7) | ≤ 0.01 | 2.00 (1.05, 3.73) | 0.04 |
| **CC** | 2 | 1.04 (0.1, 11.26) | 0.98 | 0.51 (0.03, 8.13) | 0.64 |
|  | ≥3 | 0.78 (0.07, 8.25) | 0.84 | 0.36 (0.02, 6.87) | 0.5 |

OR: Odds Ratios; CI: Confidence Interval; SLEs: Stressful Life Events

**†**Crude model; **‡**Model adjusted for age, sex, anxiety, depression, child SLEs (0/≥1)

**Table S6.** Interaction between CNR1’s rs2023239 genotype and adult SLEs (≥ 2 ^†^ or >3 ^‡^) on the odds of problematic alcohol use (i.e., AUDIT score ≥ 8)

|  | **< 2 SLE** |  | **≥ 2 SLE** |  | **OR (95% CI) for SLE within strata of genotype** |
| --- | --- | --- | --- | --- | --- |
|  | **N case/control** | **OR (95% CI)** | **N case/control** | **OR (95% CI)** |  |
| **TT** | 96 / 1,015 | 1 | 86 / 717 | 1.15 (0.83, 1.61)  p = 0.4 | 1.16 (0.83, 1.63)  p = 0.38 |
| **CC & CT** | 36 / 441 | 0.92 (0.61, 1.4)  p = 0.7 | 51 / 284 | 1.48 (0.99, 2.2)  p = 0.06 | 1.57 (0.93, 2.64)  p = 0.09 |
| **ORs (95% CI) for genotype within strata of SLE** |  | 0.93 (0.61, 1.41) p = 0.73 |  | 1.28 (0.86, 1.9)  p = 0.22 |  |

Case/control refers to problematic alcohol use (yes/no)

OR: Odds Ratios; CI: Confidence Interval; SLEs: Stressful Life Events

Estimates adjusted for age, sex, diagnosis of anxiety or depression, child SLEs (≥1)

^†^**Crude RERI estimate for adult SLEs (≥ 2) = 0.77 (95% CI: 0.07-1.47; p-value = 0.03)**

^†^**Adjusted RERI estimate for adult SLEs (≥ 2)** = **0.4 (95% CI: -0.25-1.05; p-value = 0.23)**, calculated with values from table above 1.48 - 0.92 - 1.15 + 1 = 0.4

^‡^**Crude RERI estimate for adult SLEs (≥ 3) = 0.82 (95% CI: -0.14-1.77; p-value = 0.1)**

^‡^**Adjusted RERI estimate for adult SLEs (≥ 3) = 0.51 (95% CI: -0.3-1.32; p-value = 0.22)**

**Table S7.** Overview of sex-stratified RERI estimates: additive interaction between CNR1’s rs2023239 genotype and child SLEs (≥1) / adult SLEs (≥ 2) on the odds of problematic alcohol use (cut-off in women: AUDIT score ≥ 6; cut-off in men: AUDIT score ≥ 8; see also Methods)

| **Additive interaction** | **RERI (95% CI); p-value** | **n** |
| --- | --- | --- |
| **Genotype x child SLEs (women)*** | 1.13 (0.4, 1.87); ≤ 0.01 | 1,559 |
| **Genotype x child SLEs (men)*** | 0.31 (-0.82, 1.43); 0.59 | 1,080 |
| **Genotype x adult SLEs (women)**** | 0.39 (-0.17, 0.96); 0.17 | 1,532 |
| **Genotype x adult SLEs (men)**** | 0.14 (-0.72, 1.0); 0.74 | 1,065 |

*Estimates adjusted for age, diagnosis of anxiety or depression

**Estimates adjusted for age, diagnosis of anxiety or depression, child SLEs (≥1)

**Table S8.** Interaction between CNR1’s rs2023239 genotype and adult SLEs (≥ 2 † or ≥ 3 ‡) in PART wave I on the odds of problematic alcohol use in PART wave II (i.e., AUDIT score ≥ 8)

|  | **< 2 SLE** |  | **≥ 2 SLE** |  | **OR (95% CI) for SLE within strata of genotype** |
| --- | --- | --- | --- | --- | --- |
|  | **N case/control** | **OR (95% CI)** | **N case/control** | **OR (95% CI)** |  |
| **TT** | 99 / 1,020 | 1 | 81 / 723 | 1.12 (0.8, 1.56)  p = 0.5 | 1.06 (0.76, 1.48)  p = 0.74 |
| **CC & CT** | 34 / 448 | 0.82 (0.54, 1.25)  p = 0.35 | 43 / 288 | 1.31 (0.87, 1.98)  p = 0.2 | 1.83 (1.63, 3.17)  p = 0.03 |
| **ORs (95% CI) for genotype within strata of SLE** |  | 0.83 (0.54, 1.26) p = 0.38 |  | 1.18 (0.78, 1.78)  p = 0.44 |  |

Case/control refers to problematic alcohol use (yes/no)

OR: Odds Ratios; CI: Confidence Interval; SLEs: Stressful Life Events

Estimates adjusted for age, sex, diagnosis of anxiety or depression, child SLEs (≥1)

^†^**Crude RERI estimate for adult SLEs (≥ 2) = 0.6 (95% CI: -0.03-1.23; p-value = 0.06)**

^†^**Adjusted RERI estimate for adult SLEs (≥ 2)** = **0.37 (95% CI: -0.24-0.99; p-value = 0.23)**, calculated with values from table above 1.31 - 0.82 - 1.12 + 1 = 0.37

^‡^**Crude RERI estimate for adult SLEs (≥ 3) = 0.44 (95% CI: -0.48-1.35; p-value = 0.35)**

^‡^**Adjusted RERI estimate for adult SLEs (≥ 3) = 0.25 (95% CI: -0.61-1.12; p-value = 0.57)**

**Table S9.** Number of observations for each combination of genotype and Five Factor Model personality trait

| **Trait** | **Number of observations (N)** | | | |
| --- | --- | --- | --- | --- |
|  | **TT** | **CT** | **CC** | **Total** |
| **Extraversion** | 1,538 | 630 | 43 | 2,211 |
| **Neuroticism** | 1,546 | 632 | 42 | 2,220 |
| **Conscientiousness** | 1,548 | 632 | 42 | 2,222 |
| **Openness** | 1,543 | 628 | 43 | 2,214 |
| **Agreeableness** | 1,525 | 625 | 43 | 2,193 |

**Table S10.** Logistic regression of problematic alcohol use (i.e., AUDIT score ≥8)^#^ on Five Factor Model trait z-scores

| **Trait** | **OR (95% CI) †** | **p-value** | **OR (95% CI) ‡** | **p-value** |
| --- | --- | --- | --- | --- |
| **Extraversion** | 0.9 (0.79, 1.03) | 0.11 | 0.96 (0.83, 1.1) | 0.53 |
| **Neuroticism** | 1.3 (1.14, 1.48) | ≤0.01 | 1.4 (1.22, 1.6) | ≤0.01 |
| **Conscientiousness** | 0.71 (0.63, 0.81) | ≤0.01 | 0.75 (0.66, 0.85) | ≤0.01 |
| **Openness** | 1.09 (0.95, 1.25) | 0.22 | 1.12 (0.97, 1.28) | 0.12 |
| **Agreeableness** | 0.77 (0.67, 0.89) | ≤0.01 | 0.8 (0.7, 0.93) | ≤0.01 |

^#^ Assessed at PART wave III

OR: Odds Ratios; CI: Confidence Interval; **†** Crude model; **‡** Model adjusted for age and sex

**Supplemental Figures**

**Figure S1**

**Fig. S1. Box plot of AUDIT score by rs2023239 genotype**

**Figure S2**

**Fig S2. Box plots for extraversion and conscientiousness z-scores by rs2023239 genotype**

**Figure S3**

**Fig S3. Box plots for neuroticism, agreeableness, and openness z-scores by rs2023239 genotype**

**Figure S4**

**Fig S4. Predicted mean extraversion z-scores across categories of rs2023239 genotype (TT/CT/CC) and stressful life events (0/≥1) in childhood**Error bars represent 95% CIs. Adjusted for age and sex. Interaction terms not statistically significant (CT*child SLEs: p-value=0.84; CC*child SLEs: p-value=0.2)

**Figure S5**

**Fig S5. Predicted mean neuroticism z-scores across categories of rs2023239 genotype (TT/CT/CC) and stressful life events (0/≥1) in childhood**Error bars represent 95% CIs. Adjusted for age and sex. Interaction terms not statistically significant (CT*child SLEs: p-value=0.78; CC*child SLEs: p-value=0.09)

**Figure S6**

**Fig S6. Predicted mean agreeableness z-scores across categories of rs2023239 genotype (TT/CT/CC) and stressful life events (0/≥1) in childhood**Error bars represent 95% CIs. Adjusted for age and sex. Interaction terms not statistically significant (CT*child SLEs: p-value=0.72; CC*child SLEs: p-value=0.52)
